# Further Validation of Measures of Target Detection and Stereotype Activation in the Stereotype Misperception Task

**DOI:** 10.3389/fpsyg.2020.573985

**Published:** 2020-11-05

**Authors:** Regina Reichardt, Andrew M. Rivers, Joerg Reichardt, Jeffrey W. Sherman

**Affiliations:** ^1^Department of Psychology, University of Regensburg, Regensburg, Germany; ^2^Department of Psychology, The University of British Columbia, Vancouver, BC, Canada; ^3^mbr Research GmbH, Regensburg, Germany; ^4^Department of Psychology, University of California, Davis, Davis, CA, United States

**Keywords:** implicit, stereotyping, multinomial modeling, automatic, stereotype activation

## Abstract

Previous research presented a multinomial model to estimate four latent processes (target detection, stereotype activation, stereotype application, guessing) that contribute to responses in the Stereotype Misperception Task, an indirect measure of stereotyping (Krieglmeyer and Sherman, 2012). The present research further investigates the validity of the target detection (D) and stereotype activation (SAC) parameters. To this end, the data from Experiment 2 and Experiment 4 in Krieglmeyer and Sherman (2012) were re-analyzed using a bootstrap method to investigate the robustness of the results. Furthermore, two conceptual replication studies were conducted and analyzed with the same bootstrap method. A manipulation of target distinctness influenced the D parameter as predicted. A manipulation of prime prototypicality influenced the SAC parameter as predicted. Taken together, the results support the validity of the D and SAC model parameters.

## Introduction

The Stereotype Misperception Task (SMT) is an indirect measure of stereotyping that is based on trait judgments of ambiguous targets that are preceded by primes representing social group members (Krieglmeyer and Sherman, 2012). The SMT was developed with the aim to provide a measurement tool that assesses the two main processes underlying stereotyping: stereotype activation and stereotype application. Stereotype activation is an increased accessibility of knowledge about social groups. Stereotype application is the use of this knowledge in judgment. Whereas earlier research used two different tasks to measure stereotype activation (e.g., word fragment completion task) and stereotype application (e.g., trait judgments; Gilbert and Hixon, 1991), the SMT provides estimates of these processes within a single measurement procedure. This approach has two advantages over the traditional “task-dissociation” approach (Sherman et al., 2014): first, the tasks typically used to assess stereotype activation and stereotype application differ in many procedural details that are therefore confounded with the to-be-measured construct. Second, research has shown that stereotype measures typically do not provide pure assessments of the to-be-measured construct. Measures of stereotype application necessarily also reflect stereotype activation. But even measures of stereotype activation have been shown to not only reflect activation but also the motivation and ability to control the activated stereotypes (Sherman et al., 2008). To overcome these shortcomings, Krieglmeyer and Sherman (2012) developed the SMT as a single measure of stereotyping and used a mathematical model to disentangle the degree to which stereotype activation and stereotype application contributed to the measurement outcome.

**FIGURE 1:**
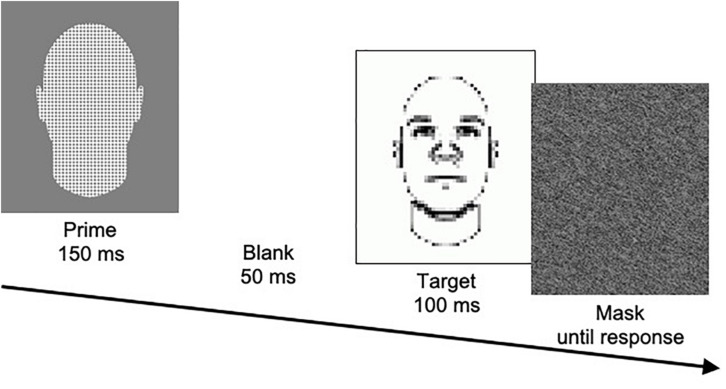
Illustration of the procedure of a trial in the SMT, showing a neutral prime and a high-threat target.

In the SMT, participants are presented with a prime picture for 150 ms, followed by a blank screen for 50 ms, followed by a target picture for 100 ms, followed by a gray pattern mask until they respond (see Figure 1). The prime is one of three types: pictures of members of two different social categories (e.g., Black men vs. White men) and pictures of neutral face-like shapes. Target pictures are blurred drawings of faces that slightly vary in facial features that are related to a particular trait (e.g., low- vs. high-threat appearance). Participants are asked to form a quick impression of the target with respect to the particular trait (e.g., whether the target appears threatening). In particular, they are asked to indicate whether the target appears more or less threatening than the average target presented in the task. When making their judgment, they are told to rely on their gut feeling. They also are told that they should attend to but not respond to the prime picture, but only judge the target picture. Participants complete a total of 144 trials across two blocks. According to the rationale of this task, stereotypes about the social categories should influence target judgments because the target is rather ambiguous and is presented very shortly. In line with this reasoning, results consistently show that targets are more frequently judged as “more threatening” when they are preceded by Black than White primes.

To disentangle the processes contributing to the target judgments, Krieglmeyer and Sherman (2012) proposed a multinomial processing tree model. Multinomial processing tree models are formal models that are used to provide estimates of latent processes that contribute to responses in a specific measurement procedure (Batchelder and Riefer, 1999). The SMT-model distinguishes four processes. Stereotype Activation (SAC) represents the extent to which the prime activates the trait stereotypically associated with the social group (e.g., threatening). Stereotype Application (SAP) represents the extent to which the activated stereotypic trait is applied to judgments of the target. Target Detection (D) represents the extent to which the objective characteristics exhibited by the target are correctly detected (e.g., high vs. low threat). Finally, Guessing (G) represents the tendency to guess that the target is more or less threatening than the average target when the other processes do not occur.

**FIGURE 2:**
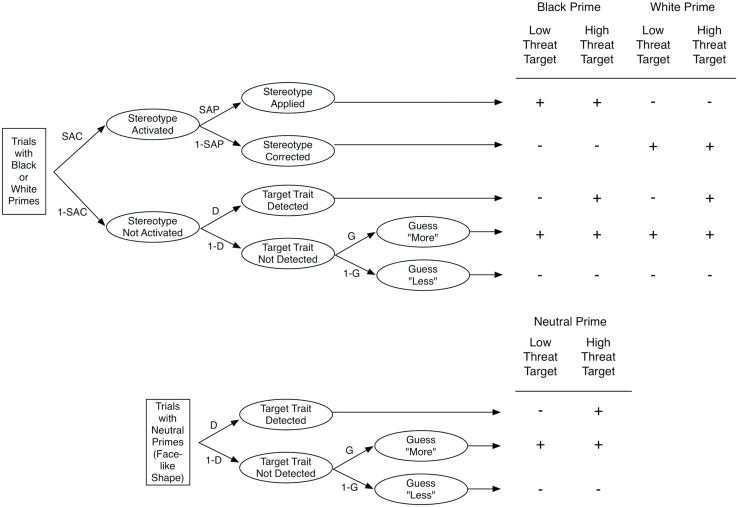
Multinomial Processing Tree Model of the SMT. The top part shows the model for Black and White primes, and the bottom part shows the model for neutral primes. The table on the right depicts the responses as a function of prime and target. The response “more threatening” is represented by a + sign and the response “less threating” is represented by a – sign. SAC, stereotype activation; 1 – SAC, lack of stereotype activation; SAP, stereotype application; 1 – SAP, stereotype correction; D, detection of target trait; 1 – D, lack of detection of target trait; G, tendency to guess “more threating; 1 – G, tendency to guess “less threating. from Krieglmeyer and Sherman (2012). Copyright 2012 by the American Psychological Association.

Figure 2 illustrates the manner in which the processes are assumed to contribute to responses. If upon presentation of a prime picture (e.g., picture of a Black man) the associated stereotype (e.g., threat) is activated (with probability SAC) and if the stereotype is applied to the judgment of the target (with probability SAP), then participants respond with “more threatening.” If the stereotype is activated but not applied (with probability SAC × 1–SAP), then participants respond with “less threatening.” If the stereotype is not activated (with probability 1–SAC) and the target trait is detected (with probability D), then participants’ response correctly reflects the appearance of the target (i.e., low vs. high threat). If the stereotype is not activated and the target trait is not detected, then participants’ response reflects a guessing tendency (with G representing the probability to guess “more threatening”).

The validity of the multinomial model of the SMT has been demonstrated in several experimental studies (Krieglmeyer and Sherman, 2012). In particular, Krieglmeyer and Sherman (2012) demonstrated both stochastic and construct validity using a *selective influence* procedure in a series of experiments. In each of these experiments, an experimental manipulation was designed that was expected *a priori* to specifically influence only one of the four multinomial process model estimates while leaving the other parameters unaffected. For instance, manipulating the extent to which the targets differed in threat appearance affected the detection parameter but not the remaining parameters. Manipulating the prototypicality of the prime affected the stereotype activation parameter but not the remaining parameters. More specifically, stereotype activation was higher for Black faces with more Afrocentric features than for Black faces with less Afrocentric features (cf. Blair et al., 2002). Building on this initial research, the SMT together with the multinomial model has been used in a growing number of studies on stereotyping (Rees et al., 2019, 2020; Rivers et al., 2019).

However, closer scrutiny of the studies in the original publication of Krieglmeyer and Sherman (2012) indicates a need to further investigate the construct validity of the detection and stereotype activation parameters. Specifically, there remains some ambiguity about the influence of target distinctness (Experiment 2) and prime prototypicality (Experiment 4) on detection and stereotype activation, respectively. These studies implemented a fully within-subjects design to investigate the impact of these manipulations on parameter estimates. To this end, two experimental stimulus sets were created with the aim that the two sets differ (i.e., low- vs. high-threat targets in Experiment 2; low- vs. high-Afrocentric Black primes in Experiment 4), whereas two other control sets were created with the aim that they do not differ (i.e., two sets with medium-threat targets in Experiment 2; two sets with White face primes and two sets with Neutral face primes in Experiment 4). In each study, each experimental stimulus set was paired with a control stimulus set.

Despite careful assignment of individual stimuli to different control stimulus sets, participants’ responses may have differed depending on the specific control set selected. This could pose a problem for the modeling analysis, because the parameter estimates might depend on the particular pairing of experimental stimuli with control stimuli.

To address these problems, we conducted bootstrap analyses on the data from Experiments 2 and 4. In particular, we created a large number of randomly chosen counterbalanced pairings of experimental stimuli with control stimuli and analyzed these data sets with the multinomial model. Thereby, we can investigate whether the results are robust across different pairings of experimental stimuli with control stimuli.

In addition, we conducted replication studies using nested within-subjects designs that avoid the problem of pre-assigning control stimuli to stimulus sets. To further investigate the robustness of the results of these studies, we applied the same bootstrap procedure to the data from these studies.

## Effects of Target Distinctness: Bootstrap Analysis of Experiment 2 From Krieglmeyer and Sherman (2012)

Experiment 2 (*N* = 35) in Krieglmeyer and Sherman (2012) sought to investigate the validity of the target detection (D) parameter by manipulating the degree of threat appearance of the targets. To this end, two target sets were created (for an overview of the design, see Table 1). The different-threat set contained target faces that were either low or high in threat appearance (i.e., two standard deviations above or below the medium-threat faces). The low-threat faces were assigned to subset A and the high-threat faces were assigned to subset B. The same-threat set contained target faces that were medium in threat appearance. Here, subset A and subset B contained the exact same faces.

**TABLE 1:** Overview of the design of Experiment 2 in Krieglmeyer and Sherman (2012).

	Target set containing different-threat targets	Target set containing same-threat targets
Target subset A	24 low-threat targets	24 medium-threat targets
Target subset B	24 high-threat targets	24 medium-threat targets

*Note that the medium-threat targets in subset A were identical to the medium-threat targets in subset B.*

Krieglmeyer and Sherman (2012) conducted a 3 (prime: White vs. neutral vs. Black) x 2 (target set: different threat vs. same threat) x 2 (target subset: A vs. B) ANOVA on relative frequencies of threat judgments and found the predicted two-way interaction between target set and target subset. In particular, participants responded more often with “more threatening” to high-threat targets than to low-threat targets (*p* = 0.017), whereas responses did not differ between the two subsets containing medium-threat targets (*p* = 0.11). However, inspection of the means revealed that responses to the two subsets with medium-threat targets were not equal (subset A: *M* = 0.38, *SD* = 0.14; subset B: *M* = 0.35, *SD* = 0.14). It is important to note that this difference could only be due to random error variance, because the two subsets contained the exact same stimuli and the order of the presentation was randomized individually for each participant.

The multinomial modeling analysis confirmed the hypotheses: the D parameter estimated from responses to low- and high-threat targets differed significantly from zero, whereas the D parameter estimated from responses to medium-threat targets did not differ significantly from zero. Furthermore, the two D parameters differed significantly.

To investigate the robustness of the results from the modeling analysis, we conducted additional modeling analyses based on a large number of randomly chosen counterbalanced pairings of low- versus high-threat faces with medium-threat faces in subsets A versus B. Thereby, we can investigate whether the results are robust across different pairings of responses to experimental stimuli with responses to control stimuli.

### Method

We created 10,000 independent and identically distributed (IDD) data sets from the raw data of Experiment 2 in Krieglmeyer and Sherman (2012), using a bootstrap approach. To create each single data set, we chose 50% of participants randomly and swapped the assignment of trials with medium-threat targets into subsets A and B. Then, frequency counts of the “more threatening” and the “less threatening” responses were aggregated across subjects for each trial type. This procedure is equivalent to the procedure of counterbalancing the medium-threat subsets A and B, with the advantage that it creates 10,000 different ways of counterbalancing across subjects.

### Results

We ran the multinomial modeling analyses on the 10,000 data sets. If the results are independent of the particular counterbalanced pairing of experimental stimuli with control stimuli, we expect that at least 95% of the data sets would confirm our hypotheses (setting Type I error rate at 5%).

First, we tested model fit for the 10,000 data sets. In particular, through maximum likelihood estimation the parameter values are estimated such that a maximum fit is reached between the response frequencies predicted by the model equations and the observed response frequencies. To test the goodness of fit, the likelihood ratio statistic *G*^2^ is computed. *G*^2^ is asymptotically chi-square distributed. A non-significant result indicates that the expected response frequencies do not significantly deviate from the observed response frequencies. The model fit the data well, with *G*^2^ falling below the critical value of 9.49 (*df* = 4), *p* > 0.05, in 98.3% of the data sets. The mean fit across all data sets was *G*^2^(4) = 4.18 (*SD* = 1.73). To further quantify model fit, we calculated the mean *w* coefficient across all data sets, which provides an estimate of the effect size of model misfit. The misfit across all data sets was very small, *w* = 0.028 (*SD* = 0.005). Mean parameter estimates from the 10,000 data sets are printed in Table 2.

**TABLE 2:** Mean parameter estimates from 10,000 bootstrapped data sets created from Experiment 2 in Krieglmeyer and Sherman (2012).

	SAC	SAP	D	G
Same threat	0.71	0.63	0.01	0.24
Different threat	0.66	0.62	0.07	0.24

Second, we tested whether the manipulation of target threat affected the D parameter but not the SAC, SAP, and G parameters. To this end, we created nested models by constraining a particular parameter, and compared the resulting *G*^2^ statistic with the *G*^2^ statistic from the baseline model in which all parameters were permitted to freely vary. A significant increase of the *G*^2^ statistic indicates that the constrained model should be rejected in favor of the baseline model.

To test whether estimates of the D parameter differ from zero, we constrained the baseline model by setting the D parameter equal to zero in the different-threat condition. As expected, this constraint significantly reduced model fit, Δ*G*^2^(1) > 3.84, *p* < 0.05, in 100% of the data sets, mean Δ*G*^2^(1) = 6.85 (*SD* < 0.01), mean *w* = 0.037 (*SD* < 0.001). Thus, D was reliably greater than zero in the different-threat condition, suggesting that participants discriminated between low- and high-threat targets. Conversely, setting the D parameter to zero in the same-threat condition did not significantly reduce model fit, Δ*G*^2^(1) < 3.84, *p* > 0.05, in 96.8% of the data sets, mean Δ*G*^2^(1) = 0.57 (*SD* = 1.23), mean *w* = 0.006 (*SD* = 0.009). Thus, D did not differ from zero in the same-threat condition, suggesting that the medium-threat targets in subsets A and B were perceived as equal in threat. To test whether the D parameter in the different-threat condition was significantly larger than the D parameter in the same-threat condition, we constrained the model by setting the two D parameters equal to each other. Surprisingly, results were not consistent across the 10,000 data sets. In 44.4% of the data sets, model fit was significantly reduced, Δ*G*^2^(1) > 3.84, *p* < 0.05, suggesting that the D parameters are different. In the remaining data sets, model fit was not significantly reduced, suggesting that the D parameters do not differ. Across all data sets, the mean statistics were: mean Δ*G*^2^(1) = 3.44 (*SD* = 1.95), mean *w* = 0.025 (*SD* = 0.009). Thus, the predicted difference between the D parameters in the same-threat versus different-threat conditions was not robust across the bootstrapped data sets.

With regard to the remaining parameters, SAC, SAP, and G responded as predicted. SAC did not differ between the same-threat and the different-threat conditions, Δ*G*^2^(1) < 3.84, *p* > 0.05 in 100% of the data sets, mean Δ*G*^2^(1) = 0.47 (*SD* = 0.04), mean *w* = 0.010 (*SD* < 0.001). SAP did not differ between the same-threat and the different-threat conditions, Δ*G*^2^(1) < 3.84, *p* > 0.05 in 100% of the data sets, mean Δ*G*^2^(1) = 0.07 (*SD* < 0.01), mean *w* = 0.004 (*SD* < 0.001). G did not differ between the same-threat and the different-threat conditions, Δ*G*^2^(1) < 3.84, *p* > 0.05 in 100% of the data sets, mean Δ*G*^2^(1) = 0.05 (*SD* = 0.06), mean *w* = 0.003 (*SD* = 0.001).

### Discussion

Across 10,000 bootstrapped data sets, results partially replicate the results from the original analyses. First, we can be confident that the model fits the data. Furthermore, we can be confident that manipulating the threat level of target faces affected only the D parameter, but not the SAC, SAP, and G parameters. More specifically, D was reliably greater than zero in the condition in which the targets differed in threat. Furthermore, D did not differ from zero in the condition in which the targets were all medium in threat. These results are consistent with the view that the D parameter reflects target detection. However, the D parameters did not reliably differ as a function of whether the targets differed in threat versus were equal in threat in a large proportion of the samples. This is surprising and inconsistent with the previous results, demonstrating D as being different from versus equal to zero in these conditions. We suspect that a floor effect contributed to this pattern because estimates of the D parameters were very small (all *D*s < 0.08). Furthermore, the mean effect size of the difference between the D parameters was very small (*w* = 0.025). Power to detect an effect of this size with *N* = 35 participants (with a total of 5040 responses, resulting from 144 trials per participant) was only 1 – β = 0.42 (G^∗^Power 3; Faul et al., 2007). To further investigate the effect of target distinctness, we conducted a conceptual replication study.

## Effects of Target Distinctness: A Conceptual Replication Study

To further investigate the impact of target distinctness on the detection parameter, we conducted a conceptual replication study. The implementation was highly similar to Experiment 2 from Krieglmeyer and Sherman (2012), with two exceptions: first, the target faces in the different-threat set were three standard deviations above or below a medium level of threat as compared to two standard deviations in the experiment from Krieglmeyer and Sherman. Thereby, we sought to increase the strength of the manipulation. Second, the target subset factor (low- vs. high-threat target) was nested within the different-threat level of the target set factor (different-threat vs. same-threat). Thereby, we avoided the problem of *a priori* assigning the medium-threat stimuli to different subsets A versus B. Finally, we increased the sample size to increase power.

### Method

#### Participants

Participants were 96 undergraduate students at the University of California, Davis who participated in the experiment for partial course credit. Data were not recorded for seven participants due to a computer malfunction. Based on previous exclusion criteria (Krieglmeyer and Sherman, 2012), we excluded two participants who self-identified as African American, as well as three participants who pressed the same key in all trials, leaving a final sample of 84 participants (19 male, 65 female). To detect an effect size of *w* = 0.0247 (as observed for the difference between the D parameters in the previous experiment) at 1 – β = 0.80 power, a total of 12866 observations is required. Thus, with 144 responses per subject, a sample size of *N* = 90 participants was required. Though our sample size was slightly smaller due to data loss and participant exclusion, we expected the power to be sufficient, given the stronger manipulation of target threat. The sample size of 84 participants (with a total of 12096 responses) was sufficient to detect an effect size of *w* = 0.0255 at 1 – β = 0.80 power.

#### Materials

Prime stimuli were the same as those used in Krieglmeyer and Sherman (2012). Target stimuli consisted of two sets of face morphs reported in Oosterhof and Todorov (2008) that were pixelated using photo-editing software. As in Krieglmeyer and Sherman, target images differed systematically in their threateningness. The different-threat target set contained 24 images that were three standard deviations above a mean level of threat and 24 images that were three standard deviations below a mean level of threat, whereas the same-threat target set contained 24 images that were at a midpoint of threat. These target images are freely available at osf.io/vp5nq.

#### Procedure

Participants completed 144 trials of the SMT procedure across two experimental blocks. The SMT procedure was identical to Krieglmeyer and Sherman (2012).

#### Design

The design was a 3 (prime: White vs. neutral vs. Black) x 2 (target set: different vs. same threat) x 2 (target subset: A/low-threat vs. B/high-threat) repeated-measures design. Target set was manipulated such that half of the trials in each block contained target threat images that were low or high in threat, and the other half of the trials contained images that were medium in threat. The Target Subset factor was nested within the different-threat level of the Target Set factor. On half of the different-threat trials, a low-threat target was presented, and on the other half of the different-threat trials, a high-threat target was presented. On the same-threat trials, a medium-threat target was sampled randomly from a single list.

For the data analysis, trials with medium-threat targets were randomly assigned to target subsets A versus B, such that all possible combinations of prime type and target subset occurred equally often. The random assignment was done individually for each participant.

### Results

#### ANOVA on the Proportion of “More Threatening” Responses

We subjected the proportion of “more threatening” responses to a 3 (prime: White vs. neutral vs. Black) x 2 (target set: different threat vs. same threat) x 2 (target subset: subset A vs. subset B) ANOVA for repeated measures (see Table 3 for descriptive statistics). The ANOVA yielded a main effect of target set, *F*(1,83) = 16.56, *p* < 0.001, $\eta_{p}^{2}$ = 0.17, and a main effect of target subset, *F*(1,83) = 30.94, *p* < 0.001, $\eta_{p}^{2}$ = 0.27, that were further qualified by the predicted interaction between target set and target subset, *F*(1,83) = 24.58, *p* < 0.001, $\eta_{p}^{2}$ = 0.23. *Post hoc* tests (Bonferroni) indicated that in the different-threat condition, threat responses were more frequent in the subset with high-threat targets than in the subset with low-threat targets, *p* < 0.001. In the same-threat condition, however, the proportion of threat responses did not differ between the subsets, *p* = 0.421.

**TABLE 3:** Mean proportion of threat judgments as a function of prime (White vs. Neutral versus Black), target set (Same vs. Different Threat), and target subset (Subset A vs. Subset B) in the conceptual replication study on target distinctness.

	White prime		Neutral prime		Black prime	
	Subset A	Subset B	Subset A	Subset B	Subset A	Subset B
Same threat	0.35 (0.20)	0.37 (0.22)	0.30 (0.24)	0.29 (0.26)	0.45 (0.27)	0.46 (0.26)
Different threat	0.31 (0.22)	0.39 (0.25)	0.27 (0.27)	0.50 (0.30)	0.44 (0.28)	0.51 (0.26)

*In the Different-Threat Condition, Subset A contained low-threat targets and Subset B contained high-threat targets. Standard deviations are printed in parentheses.*

Furthermore, the ANOVA yielded the predicted main effect of prime, *F*(2,166) = 10.35, *p* < 0.001, $\eta_{p}^{2}$ = 0.11. *Post hoc* tests (Bonferroni) indicated that Black primes led to more threat judgments than White primes, *p* = 0.002, or neutral primes, *p* < 0.001. White primes did not differ from neutral primes, *p* > 0.999.

Furthermore, the ANOVA yielded significant two-way interactions of prime and target set, *F*(2,166) = 11.45, *p* < 0.001, $\eta_{p}^{2}$ = 0.12, prime and target subset, *F*(2,166) = 7.62, *p* = 0.001, $\eta_{p}^{2}$ = 0.08, as well as a significant three-way interaction of prime, target set, and target subset, *F*(2,166) = 12.18, *p* < 0.001, $\eta_{p}^{2}$ = 0.13. *Post hoc* tests (Bonferroni) indicated that threat responses were more frequent to high- as compared to low-threat targets after White primes, *p* = 0.024, after neutral primes, *p* < 0.001, and after Black primes, *p* = 0.008. Conversely, threat responses did not differ between subsets in the same threat level condition after White primes, *p* = 0.348, after neutral primes, *p* = 0.686, and after Black primes, *p* = 0.557.

### Modeling Results

We first tested the model on the response frequencies aggregated from the raw data (see Table 4 for parameter estimates). This experiment already has a built-in random assignment of medium-threat stimuli to target subsets A and B, rendering it unlikely that the modeling results are due to the particular pairing of stimuli. Nevertheless, the bootstrap method applied to Experiment 2 from Krieglmeyer and Sherman (2012) provides additional confidence in the robustness of results, as it is based on 10,000 random assignments. Therefore, we additionally implemented this bootstrap procedure.

**TABLE 4:** Parameter estimates in the conceptual replication study on target distinctness.

	SAC	SAP	D	G
Same threat	0.55 [0.46,0.63]	0.59 [0.56,0.62]	0.01 [−0.03,0.04]	0.29 [0.27,0.31]
Different threat	0.43 [0.31,0.55]	0.64 [0.59,0.69]	0.19 [0.15,0.23]	0.34 [0.31,0.36]

*95% confidence intervals are printed in brackets.*

The model did not fit the data well in traditional terms, *G*^2^(4) = 13.11, *p* = 0.01, yet the extent of misfit was small, *w* = 0.033. The manipulation of target set (different vs. same threat) affected the D parameter as expected. D was greater than zero in the different-threat condition, Δ*G*^2^(1) = 126.13, *p* < 0.001, *w* = 0.102. Conversely, D did not differ from zero in the same-threat condition, Δ*G*^2^(1) = 0.08, *p* = 0.78, *w* = 0.003 Moreover, the D parameters in the same- threat versus different-threat conditions were significantly different, Δ*G*^2^(1) = 54.48, *p* < 0.001, *w* = 0.067. D was higher when the targets differed in threat as compared to when they were equal in threat.

As expected, SAC did not differ between target set conditions, Δ*G*^2^(1) = 2.64, *p* = 0.104, *w* = 0.015, and SAP did not differ between target set conditions, Δ*G*^2^(1) = 3.06, *p* = 0.080, *w* = 0.016. However, G differed between target set conditions, Δ*G*^2^(1) = 7.42, *p* = 0.006, *w* = 0.025. When targets were medium in threat, participants exhibited a stronger tendency to guess “less threatening” compared to when targets were low or high in threat.

We applied the same bootstrap procedure as for the data from Experiment 2 in Krieglmeyer and Sherman (2012). In particular, we created 10,000 bootstrapped data sets by swapping the assignment of medium-threat trials to target subsets A versus B for 50% of participants who were randomly chosen (see Table 5 for mean parameter estimates across all data sets). The model did not fit the data well in traditional terms, *G*^2^(4) > 9.49, *p* < 0.05, in 100% of the data sets, mean *G*^2^(4) = 13.80 (*SD* = 1.80). Yet, the extent of misfit was small, mean *w* = 0.034 (*SD* = 0.002).

**TABLE 5:** Mean parameter estimates from 10,000 bootstrapped data sets in the conceptual replication study on target distinctness.

	SAC	SAP	D	G
Same threat	0.55	0.59	0.01	0.29
Different threat	0.43	0.64	0.19	0.34

As expected, D was reliably greater than zero in the different-threat condition, Δ*G*^2^(1) > 3.84, *p* < 0.05, in 100% of the data sets, mean Δ*G*^2^(1) = 126.13 (*SD* < 0.01), mean *w* = 0.102 (*SD* < 0.001). Conversely, D did not differ from zero in the same threat levels condition, Δ*G*^2^(1) < 3.84, *p* > 0.05, in 99.5% of the data sets, mean Δ*G*^2^(1) = 0.30 (*SD* = 0.66), mean *w* = 0.003 (*SD* = 0.004). Moreover, the D parameters in the same-threat versus different-threat conditions were reliably different, Δ*G*^2^(1) > 3.84, *p* < 0.05, in 100% of the data sets, mean Δ*G*^2^(1) = 58.63 (*SD* = 8.18), mean *w* = 0.069 (*SD* = 0.005).

As expected, SAC did not differ between the same-threat and the different-threat conditions, Δ*G*^2^(1) < 3.84, *p* > 0.05, in 100% of the data sets, mean Δ*G*^2^(1) = 2.67 (*SD* = 0.05), mean *w* = 0.015 (*SD* < 0.001). Furthermore, SAP did not differ between the same-threat and the different-threat conditions, Δ*G*^2^(1) < 3.84, *p* > 0.05, in 100% of the data sets, mean Δ*G*^2^(1) = 3.09 (*SD* = 0.03), mean *w* = 0.016 (*SD* < 0.001). However contrary to expectations, the G parameter differed between the same-threat and the different-threat condition, Δ*G*^2^(1) > 3.84, *p* < 0.05, in 100% of the data sets, mean Δ*G*^2^(1) = 7.65 (*SD* = 0.39), mean *w* = 0.025 (*SD* < 0.001). When targets were medium in threat, participants showed a stronger tendency to guess “less threatening” compared to when targets were low or high in threat. In sum, results from the bootstrap analysis perfectly replicate results from the analysis of the original data set.

### Discussion

Most importantly, hypothesis tests support the validity of the D parameter: the manipulation of target threat reliably affected the D parameter as predicted. When the targets differed in threat, D was higher than zero. When the targets did not differ in threat, D was equal to zero. Furthermore, D was higher when the targets differed in threat than when they were equal in threat. Probably due to the stronger manipulation of target threat distinctness (three instead of two standard deviation as in Experiment 2 from Krieglmeyer and Sherman, 2012), the size of this effect was larger (*w* = 0.067) than in Experiment 2 from Krieglmeyer and Sherman (*w* = 0.025).

Supporting the discriminant validity of the D parameter, the manipulation of target threat did not affect the SAC and SAP parameters. However, surprisingly, the manipulation of target threat affected the G parameter. When targets were medium in threat, participants exhibited a tendency to guess “less threatening” compared to when targets were low or high in threat. Though this effect was small (*w* = 0.025), further research is needed to elucidate this finding.

The bootstrap analysis perfectly replicates the modeling results from the original data set. This indicates that the nested design together with the procedure of randomly assigning medium-threat trials to target subsets A and B sufficiently minimizes potential biases resulting from the pairing of experimental stimuli with control stimuli.

In sum, the results confirm the validity of the D parameter reflecting target trait detection.

## Effects of Prime Prototypicality: Bootstrap Analysis of Experiment 4 From Krieglmeyer and Sherman (2012)

Experiment 4 (*N* = 32) in Krieglmeyer and Sherman (2012) sought to investigate the validity of the stereotype activation (SAC) parameter. Based on research showing that manipulations of prime prototypicality affect stereotype activation but not stereotype control (Blair et al., 2002, 2004), the authors predicted that manipulating the prototypicality of the Black primes would affect only the SAC parameter but not the remaining parameters.

To test this hypothesis, the experiment presented 12 low versus 12 high Afrocentric Black prime pictures. The White and neutral prime pictures were the same as in previous experiments. Based on pilot data, the White primes were assigned to prime sets A and B, such that the two sets did not differ in mean threat. The neutral primes were randomly assigned to prime sets A and B. All primes were presented in random order. For the data analysis, the prime set A was paired with the low Afrocentric Black faces and the prime set B was paired with the high Afrocentric Black faces (see Table 6 for an overview of the design), resulting in a 3 (prime: White vs. neutral vs. Black) x 2 (prime set: A/low Afrocentric Black faces vs. B/high Afrocentric Black faces) x 2 (target: low vs. high threat) repeated measures design. Modeling results showed that the degree of Afrocentric features of Black primes increased the SAC parameter but did not affect the remaining parameters.

**TABLE 6:** Overview of the design of Experiment 4 in Krieglmeyer and Sherman (2012).

	White primes	Neutral primes	Black primes
Prime Set A	12 White Faces	12 Neutral Faces	12 Low-Afrocentric Black Faces
Prime Set B	12 White Faces	12 Neutral Faces	12 High-Afrocentric Black Faces

Despite careful assignment of the White and neutral pictures to prime sets A and B, participants’ responses to White and neutral primes might nevertheless differ between prime sets, producing a potential confound. In fact, the ANOVA on the threat judgments surprisingly revealed that the White faces in prime set B were judged more threatening than the White faces in prime set A (*p* = 0.046). Likewise, the neutral faces in prime set B were judged more threatening than the neutral faces in prime set A (*p* = 0.014). This is particularly problematic for the modeling analysis, because the SAC and SAP parameters are estimated from responses to both Black and White primes. As a consequence, changes in SAC and SAP could result from differences between low versus high Afrocentric Black primes and/or from differences between White primes in prime set A versus B.

To address this possibility, we analyzed the data from Experiment 4 in Krieglmeyer and Sherman (2012) using a bootstrap procedure based on a large number of randomly chosen counterbalanced pairings of White and neutral prime sets A versus B with low versus high Afrocentric Black primes. Thereby, we can investigate whether the results are independent of potential differences between White or neutral stimuli in prime sets A versus B.

### Method

We created 10,000 bootstrapped data sets from the raw data of Experiment 4 in Krieglmeyer and Sherman (2012). To create each single data set, we chose 50% of participants randomly and swapped the assignment of White prime stimuli to sets A and B. Then, we chose another 50% of participants randomly and swapped the assignment of Neutral prime stimuli to sets A and B. Then, frequency counts of the “more threatening” and the “less threatening” responses were aggregated across subjects for each trial type. This procedure is equivalent to the procedure of counterbalancing prime sets A and B, with the advantage that it creates 10,000 different ways of counterbalancing across subjects.

### Results

We ran the multinomial modeling analyses on the 10,000 data sets. The model fit the data well, with *G*^2^ falling below the critical value of 9.49 (*df* = 4), *p* > 0.05, in 99.3% of the data sets, mean *G*^2^(4) = 2.79 (*SD* = 1.68), mean *w* = 0.024 (*SD* = 0.006). Mean parameter estimates from the 10,000 data sets are shown in Table 7.

**TABLE 7:** Mean parameter estimates from 10,000 data sets created from Experiment 4 in Krieglmeyer and Sherman (2012).

	SAC	SAP	D	G
Prime set A (Low Afrocentric)	0.44	0.66	0.07	0.26
Prime set B (High Afrocentric)	0.95	0.70	0.09	0.25

As expected, SAC was reliably higher for prime set B, which contained high-Afrocentric Black faces than for prime set A, which contained low-Afrocentric Black faces, Δ*G*^2^(1) > 3.84, *p* < 0.05, in 100% of the data sets, mean Δ*G*^2^(1) = 41.22 (*SD* = 9.19), mean *w* = 0.094 (*SD* = 0.011).

Regarding SAP, the results were less consistent across the 10,000 data sets. The hypothesis that SAP does not differ between prime sets was supported in 82.3% of the data sets, Δ*G*^2^(1) < 3.84, *p* > 0.05. In the remaining data sets, SAP differed significantly as a function of prime set, Δ*G*^2^(1) > 3.84, *p* < 0.05. As can be seen in Table 7, across all data sets SAP was higher for high-Afrocentric Black faces than for low-Afrocentric Black faces. The mean statistics across all data sets were: mean Δ*G*^2^(1) = 2.05 (*SD* = 2.46), mean *w* = 0.017 (*SD* = 0.012).

With respect to D and G, results were reliable across data sets: D did not differ between prime sets in 99.4% of the data sets, mean Δ*G*^2^(1) = 0.60 (*SD* = 0.76), mean *w* = 0.009 (*SD* = 0.007). G did not differ between prime sets in 99.8% of the data sets, mean Δ*G*^2^(1) = 0.47 (*SD* = 0.62), mean *w* = 0.008 (*SD* = 0.006).

### Discussion

Most importantly, the bootstrap analysis demonstrated that the effect of prototypicality on SAC is independent of the particular pairing of White and Neutral prime stimuli with low and high Afrocentric Black prime stimuli. Thus, we can be confident that prototypicality increases the SAC parameter, supporting the validity of this parameter as reflecting stereotype activation. Furthermore, we can be confident that manipulating the prototypicality of Black prime faces did not affect the D and G parameters, supporting the discriminant validity. With respect to SAP, results were less clear. Prototypicality did not influence SAP in 82.3% of the data sets, while it increased SAP in the remaining data sets. Thus, the influence of prototypicality on SAP depends to some extent on the particular pairing of low versus high Afrocentric Black faces with White and neutral stimuli. Because these results do not allow clear-cut conclusions regarding SAP, we conducted a conceptual replication study.

## Effects of Prime Prototypicality: A Conceptual Replication Study

To further investigate the impact of prime prototypicality on stereotype activation and stereotype application, we conducted a conceptual replication study. The prime and target stimuli were identical to Experiment 4 in Krieglmeyer and Sherman (2012). Different from the design of Experiment 4, Black prototypicality (low vs. high Afrocentric Black faces) was nested within the Black level of the prime factor. Thereby, we avoid the problem of *a priori* assigning the White and Neutral prime stimuli to different sets. Furthermore, we increased sample size to increase power. Finally, we manipulated the interstimulus interval between prime offset and target onset within-subjects. In particular, on half of the trials the interstimulus interval was short (ISI = 0 ms), whereas on the other half of the trials the interstimulus interval was long (ISI = 175). Because we did not find clear-cut results with respect to SAP in the previous experiment with ISI = 50 ms, we decreased ISI to 0 ms. We reasoned that with a shorter ISI the likelihood of controlled processing may be reduced (cf. Rivers et al., 2019), thereby providing better conditions to isolate the effects of prototypicality on stereotype activation. Because of the additional ISI-manipulation, the number of trials was increased to 216.

### Method

#### Participants

Participants were 92 undergraduate students at the University of California, Davis who participated for partial course credit. Two participants who self-identified as African American were excluded, leaving a final sample of 90 participants (7 male, 83 female). A sample of 90 participants (with a total of 9720 responses, resulting from 108 responses per participant per ISI condition) provides 1 – β = 0.99 power to detect an effect size of *w* = 0.094 (as observed for the difference between the SAC parameters in Experiment 4 from Krieglmeyer and Sherman, 2012).

#### Materials

The stimuli were the same as those used in Experiment 4 in Krieglmeyer and Sherman (2012). In particular, prime stimuli were 24 White faces, 12 low Afrocentric Black faces, 12 high Afrocentric Black faces, and 24 neutral face-like shapes. Target stimuli were 24 low-threat drawings and 24 high-threat drawings (i.e., two standard deviations above and below a mean level of threat).

#### Procedure

Participants completed 216 trials of the SMT procedure across three experimental blocks. The SMT procedure was identical to Krieglmeyer and Sherman (2012), with the only exception that the interstimulus interval (ISI) between prime and target was manipulated within-subjects. On half of the trials, the interstimulus interval was short (ISI = 0 ms), whereas on the other half of the trials the interstimulus interval was long (ISI = 175 ms). All trials were presented in random order, with the constraint that all trial types occurred equally often in each block.

#### Design

The design was a 3 (prime: White vs. Neutral vs. Black) x 2 (prime set: A/low Afrocentric vs. B/high Afrocentric) x 2 (target: low-threat vs. high-threat) x 2 (ISI: short vs. long) repeated-measures design. The prime set factor (low vs. high Afrocentric Black faces) was nested within the Black level of the prime factor. On half of the Black prime trials, a low Afrocentric Black face was presented, whereas on the other half of the Black prime trials a high Afrocentric Black face was presented. On White prime trials, the White images were sampled randomly from a single list of all White images. The same random sampling procedure was used for the neutral prime trials.

For the data analysis, trials with White primes were randomly assigned to Prime Sets A versus B, such that all possible combinations of Prime Set, Target, and ISI occurred equally often. The same procedure was used for neutral prime trials. The random assignment was done individually for each participant.

### Results

#### ANOVA on the Proportion of “More Threatening” Responses

We subjected the proportion of “more threatening” responses to a 3 (prime: White vs. neutral vs. Black) x 2 (prime set: A/low-Afrocentric vs. B/high-Afrocentric) x 2 (target: low vs. high threat) x 2 (ISI: short vs. long) ANOVA for repeated measures.

The analysis revealed significant main effects of prime, *F*(2,178) = 14.88, *p* < 0.001, $\eta_{p}^{2}$ = 0.14, and prime set, *F*(1,89) = 104.15, *p* < 0.001, $\eta_{p}^{2}$ = 0.54, that were qualified by the predicted interaction between prime and prime set, *F*(2,178) = 79.33, *p* < 0.001, $\eta_{p}^{2}$ = 0.47. *Post hoc* tests (Bonferroni) indicated that high Afrocentric Black primes led to more threat judgments than low Afrocentric primes, *p* < 0.001 (see Table 8). In contrast, threat judgments following White and neutral primes did not differ between prime sets, *p*s > 0.5. Furthermore, high-Afrocentric Black primes led to more threat judgments than White primes in prime set B, *p* < 0.001, and neutral primes in prime set B, *p* < 0.001, while White primes did not differ from neutral primes in prime set B, *p* > 0.999. Conversely, threat judgments did not differ after low-Afrocentric Black primes compared to White primes in prime set A, *p* = 0.118, and compared to neutral primes in prime set A, *p* = 0.099. Also, White primes did not differ from neutral primes in prime set A, *p* > 0.999.

**TABLE 8:** Mean proportion of threat judgments as a function of prime (White vs. Neutral vs. Black), target (Low vs. High Threat), prime set (Low vs. High Afrocentric Black Primes), and interstimulus interval (Short vs. Long) in the conceptual replication study on prototypicality.

	White prime		Neutral prime		Black prime	
	Low threat	High threat	Low threat	High threat	Low threat	High threat
**Short interstimulus interval**						
Prime set A (Low Afrocentric)	0.30 (0.25)	0.33 (0.25)	0.29 (0.32)	0.34 (0.33)	0.38 (0.30)	0.43 (0.30)
Prime set B (High Afrocentric)	0.29 (0.25)	0.33 (0.25)	0.28 (0.31)	0.37 (0.33)	0.60 (0.31)	0.61 (0.31)
**Long interstimulus interval**						
Prime set A (Low Afrocentric)	0.35 (0.22)	0.36 (0.25)	0.29 (0.32)	0.37 (0.33)	0.42 (0.30)	0.43 (0.31)
Prime set B (High Afrocentric)	0.35 (0.25)	0.37 (0.25)	0.29 (0.31)	0.37 (0.34)	0.61 (0.31)	0.64 (0.32)

*Standard deviations are printed in parentheses.*

Furthermore, the ANOVA revealed a significant main effect of target threat, *F*(1,89) = 12.58, *p* = 0.001, $\eta_{p}^{2}$ = 0.12, that was qualified by a significant interaction of target threat and prime, *F*(2,178) = 5.99, *p* = 0.003, $\eta_{p}^{2}$ = 0.06. *Post hoc* tests (Bonferroni) indicated that the effect of target threat was significant after neutral primes, *p* < 0.001, but not after White primes, *p* = 0.072, and Black primes, *p* = 0.053. Differences between prime types were comparable for both low- and high-threat targets, with significant differences between White and Black primes (*p*s < 0.001), and Black and neutral primes (*p*s < 0.002), but not between White and neutral primes (*p*s = 1). Finally, the ANOVA revealed a significant main effect of ISI, *F*(1,89) = 19.72, *p* < 0.001, $\eta_{p}^{2}$ = 0.18, indicating that the proportion of threat judgments was higher when ISI was longer. No other effects were significant, *F*s < 2.8, *p*s > 0.06.

#### Modeling Results

We analyzed the data separately for the short and long ISI condition. Again, we first modeled the original data set (see Table 9 for parameter estimates), and then applied the bootstrap procedure to further investigate the robustness of the results.

**TABLE 9:** Parameter estimates in the conceptual replication study on prime prototypicality.

	SAC	SAP	D	G
**Short interstimulus interval**				
Prime set A (Low Afrocentric)	0.24 [0.11,0.37]	0.69 [0.57,0.80]	0.05 [0.02,0.08]	0.31 [0.28,0.33]
Prime set B (High Afrocentric)	0.75 [0.66,0.85]	0.70 [0.66,0.73]	0.09 [0.05,0.13]	0.31 [0.28,0.33]
**Long interstimulus interval**				
Prime set A (Low Afrocentric)	0.37 [0.24,0.50]	0.59 [0.54,0.65]	0.05 [0.02,0.09]	0.32 [0.29,0.34]
Prime set B (High Afrocentric)	0.95 [0.86, 1.05]	0.64 [0.62,0.66]	0.08 [0.04,0.13]	0.32 [0.29,0.34]

*95% confidence intervals appear in brackets.*

In the short ISI condition, the model fit the data well, *G*^2^(4) = 1.34, *p* = 0.854, *w* = 0.012. The manipulation of Black prototypicality affected all parameters as expected. In particular, SAC was higher for the prime set containing high-Afrocentric Black primes than for the prime set containing low-Afrocentric Black primes, Δ*G*^2^(1) = 42.45, *p* < 0.001, *w* = 0.066. SAP did not differ between prime sets, Δ*G*^2^(1) < 0.01, *p* = 0.959, *w* < 0.001. D did not differ between prime sets, Δ*G*^2^(1) = 2.34, *p* = 0.126, *w* = 0.016. G did not differ between prime sets, Δ*G*^2^(1) < 0.01, *p* = 0.954, *w* < 0.001.

In the long ISI condition, the model fit the data well, *G*^2^(4) = 3.78, *p* = 0.436, *w* = 0.020. The manipulation of Black prototypicality affected all parameters as expected. In particular, SAC was higher for the prime set containing high-Afrocentric Black primes than for the prime set containing low-Afrocentric Black primes, Δ*G*^2^(1) = 55.78, *p* < 0.001, *w* = 0.076. SAP did not differ between prime sets, Δ*G*^2^(1) = 1.54, *p* = 0.215, *w* = 0.013. D did not differ between prime sets, Δ*G*^2^(1) = 0.92, *p* = 0.338, *w* = 0.010. G did not differ between prime sets, Δ*G*^2^(1) < 0.01, *p* = 0.929, *w* < 0.001.

Using the same bootstrap approach as for Experiment 4 from Krieglmeyer and Sherman (2012), we created 10,000 data sets by swapping the assignment of White and neutral stimuli, respectively, to prime sets A versus B for 50% of randomly chosen participants. For each data set, we first swapped the assignment to prime sets, and then split the data as a function of the ISI condition. Thus, any potential differences between the short and long ISI condition cannot be due to the particular selection of participants for swapping. Mean parameter estimates across all data sets are shown in Table 10.

**TABLE 10:** Mean parameter estimates from 10,000 bootstrapped data sets in the conceptual replication study on prime prototypicality.

	SAC	SAP	D	G
**Short interstimulus interval**				
Prime set A (Low Afrocentric)	0.22	0.72	0.06	0.31
Prime set B (High Afrocentric)	0.76	0.69	0.07	0.31
**Long interstimulus interval**				
Prime set A (Low Afrocentric)	0.38	0.59	0.06	0.32
Prime set B (High Afrocentric)	0.94	0.64	0.08	0.32

In the short ISI condition, the model fit the data well, *G*^2^(4) < 9.49, *p* > 0.05, in 99.4% of the data sets, mean *G*^2^(4) = 1.92 (*SD* = 1.91), mean *w* = 0.012 (*SD* = 0.006). The manipulation of Black prototypicality affected all parameters as expected. In particular, SAC was higher for the prime set containing high-Afrocentric Black primes than for the prime set containing low-Afrocentric Black primes, Δ*G*^2^(1) > 3.84, *p* < 0.05, in 100% of the data sets, mean Δ*G*^2^(1) = 46.14 (*SD* = 9.33), mean *w* = 0.069 (*SD* = 0.007). SAP did not differ between prime sets, Δ*G*^2^(1) < 3.84, *p* > 0.05, in 97.6% of the data sets, mean Δ*G*^2^(1) = 0.78 (*SD* = 1.05), mean *w* = 0.007 (*SD* = 0.005). D did not differ between prime sets, Δ*G*^2^(1) < 3.84, *p* > 0.05, in 98.1% of the data sets, mean Δ*G*^2^(1) = 0.80 (*SD* = 1.01), mean *w* = 0.007 (*SD* = 0.005). G did not differ between prime sets, Δ*G*^2^(1) < 3.84, *p* > 0.05, in 97.9% of the data sets, mean Δ*G*^2^(1) = 0.75 (*SD* = 1.03), mean *w* = 0.007 (*SD* = 0.005).

In the long ISI condition, the model fit the data well, *G*^2^(4) < 9.49, *p* > 0.05, in 97.3% of the data sets, mean *G*^2^(4) = 4.12 (*SD* = 2.14), mean *w* = 0.020 (*SD* = 0.005). The manipulation of Black prototypicality was reliable across data sets for SAC and G, but less so for D, and not at all reliable for SAP. In particular, SAC was higher for the prime set containing high-Afrocentric Black primes than for the prime set containing low-Afrocentric Black primes, Δ*G*^2^(1) > 3.84, *p* < 0.05, in 100% of the data sets, mean Δ*G*^2^(1) = 52.89 (*SD* = 9.53), mean *w* = 0.073 (*SD* = 0.007). SAP did not differ between prime sets, Δ*G*^2^(1) < 3.84, *p* > 0.05, in 60.3% of the data sets. In the remaining data sets, SAP was significantly higher when Black primes were high-Afrocentric as compared to low-Afrocentric. Across all data sets the mean statistics were: mean Δ*G*^2^(1) = 3.74 (*SD* = 3.20), mean *w* = 0.018 (*SD* = 0.009). D did not differ between prime sets, Δ*G*^2^(1) < 3.84, *p* > 0.05, in 94.7% of the data sets, mean Δ*G*^2^(1) = 1.17 (*SD* = 1.35), mean *w* = 0.009 (*SD* = 0.006). G did not differ between prime sets, Δ*G*^2^(1) < 3.84, *p* > 0.05, in 99.5% of the data sets, mean Δ*G*^2^(1) = 0.48 (*SD* = 0.67), mean *w* = 0.006 (*SD* = 0.004).

### Discussion

In the short ISI condition, results are clear-cut. The model fit the data and Black prototypicality affected the SAC parameter but not the remaining parameters. These results replicate across 10,000 bootstrapped data sets, confirming the reliability of the findings. Thus, we can clearly show that these results are independent of the particular pairing of White and Neutral prime stimuli with low- versus high-Afrocentric Black faces.

In the long ISI condition, results are partially different. Consistent with the results from the short ISI condition, the model fit the data and Black prototypicality increased the SAC parameter. Furthermore, the G parameter was not affected by prototypicality. Different from the short ISI condition, results on the D parameter were somewhat less reliable than expected. D was not affected by Black prototypicality in 94.7% of the data sets. With respect to SAP, results were unreliable: black prototypicality increased the SAP parameter in about 40% of the data sets, whereas it did not affect SAP in about 60% of the data sets. Thus, unequivocal conclusions about the impact of Black prototypicality on SAP are not possible in this condition.

In sum, the results from this study confirm the validity of the model parameters in the short ISI condition. In particular, Black prototypicality selectively influenced SAC, supporting the validity of this parameter as reflecting stereotype activation. The remaining parameters were unaffected by Black prototypicality, providing evidence for discriminant validity. Thus, the SMT-procedure with ISI = 0 ms provides a reliable measurement tool for investigating the selective influence of prime prototypicality on stereotype activation.

## General Discussion

The present research investigated the reliability of the impact of two different manipulations (i.e., target distinctness and prime prototypicality) on SMT modeling parameters. Based on the present research we can draw the following conclusions.

(1) When investigating the impact of stimulus manipulations on model parameters, we advise against pre-assigning control stimuli to different stimulus sets that are then paired with experimental stimulus sets. Even careful pretesting of the control stimuli does not guarantee that the control stimulus sets are not perceived differently, which can cause distortions in the modeling analyses. To minimize this potential source of error, we recommend assigning the control stimuli to stimulus sets randomly for each individual participant. The present results show that this procedure led to reliable results that were consistent with the results from the bootstrap analysis in the Experiment on target distinctness as well as in the experiment on prime prototypicality (except for the long ISI condition).

(2) The present results confirm the validity of the target detection (D) parameter. A manipulation of target threat distinctness has been shown to reliably influence the D parameter. D increases with target faces differing in threat appearance, suggesting that D reflects the detection of the target trait.

(3) The present results confirm the validity of the stereotype activation (SAC) parameter. A manipulation of prime prototypicality has been shown to reliably influence the SAC parameter. SAC increases with Black faces exhibiting more Afrocentric facial features, suggesting that SAC reflects the activation of stereotypes about Black people.

(4) With a short interstimulus interval between prime offset and target onset (ISI = 0 ms), the manipulation of prime prototypicality did not influence the stereotype application (SAP) parameter. With a long interstimulus interval (ISI = 175 ms), results on SAP were not reliable. Therefore, we recommend implementing ISI = 0 ms, if one wants to investigate the selective influence of prototypicality on stereotype activation.

### Future Directions

There are several avenues for future research. First, it would be interesting to elucidate why we did not find reliable results on the impact of prime prototypicality on the stereotype application parameter at a longer time interval. Although this is not central to the purpose of the present research, it is nevertheless surprising and warrants further investigation.

Second, it would be interesting to study how different manipulations affect the model parameters as a function of the time available for stimulus processing and/or responding. For instance, previous research has shown that stereotype activation increases with longer time intervals in the SMT, because prime pictures are processed more thoroughly (Rivers et al., 2019). Conversely, stereotype application decreases with longer time intervals in the SMT, possibly because people are better able to control unwanted influences of activated stereotypes on judgments when more time is available. In a similar vein, several studies using other priming tasks have shown that conscious as compared to unconscious prime processing instigates regulatory processes that result in prime-inconsistent responses (Lapate et al., 2014, 2016; Ponsi et al., 2017). Even though in the present task prime stimuli are not presented outside conscious awareness, a longer processing time may nevertheless increase the likelihood of regulatory processes taking place. Furthermore, additional manipulations such as prime prototypicality may lead to complex interactions. For instance, the pattern of parameter estimates in the present experiment on prime prototypicality suggests that the decrease in stereotype application with more time is more pronounced for low-Afrocentric than for high-Afrocentric Black primes. This may suggest that with more time available, people are more likely to control stereotype application for low-prototypic group members than for high-prototypic group members. Although speculative, people may feel more licensed to apply stereotypes when presented with high-prototypic as compared to low-prototypic group members. Alternatively, or in addition, people may lack the ability to control stereotyping when confronted with high-prototypic group members (Blair et al., 2004). Provided reliable estimates of SAP, it would be interesting to further investigate this pattern.

Finally, more research is needed on the validity of the guessing (G) and the stereotype application (SAP) parameters. It would be worthwhile to replicate and extend the original studies from Krieglmeyer and Sherman (2012) on these parameters.

## Conclusion

The present research provides further evidence on the validity of the target detection (D) and the stereotype activation (SAC) parameters. A manipulation of target distinctness confirmed the validity of the D parameter as reflecting target detection. A manipulation of prime prototypicality confirmed the validity of the SAC parameter as reflecting stereotype activation. Bootstrap analyses bolstered the reliability of these findings.

## Data Availability Statement

The raw data of Experiments 2 and 4 from Krieglmeyer and Sherman (2012) are available at osf.io/cp9ga/. The raw data of the conceptual replication studies as well as the results from all bootstrap analyses are available at osf.io/r8w2z/.

## Ethics Statement

The studies involving human participants were reviewed and approved by IRB UC Davis. The participants provided their written informed consent to participate in this study.

## Author Contributions

RR, AR, and JS wrote the manuscript and designed the studies. RR, AR, and JR did the analyses. All authors contributed to the article and approved the submitted version.

## Conflict of Interest

JR was employed by the company mbr research GmbH. The remaining authors declare that the research was conducted in the absence of any commercial or financial relationships that could be construed as a potential conflict of interest.
